# Repeated Vaccination of Cows with HIV Env gp140 during Subsequent Pregnancies Elicits and Sustains an Enduring Strong Env-Binding and Neutralising Antibody Response

**DOI:** 10.1371/journal.pone.0157353

**Published:** 2016-06-14

**Authors:** Behnaz Heydarchi, Rob J. Center, Christopher Gonelli, Brian Muller, Charlene Mackenzie, Georges Khoury, Marit Lichtfuss, Grant Rawlin, Damian F. J. Purcell

**Affiliations:** Department of Microbiology and Immunology, at The Peter Doherty Institute for Infection & Immunity, The University of Melbourne, Melbourne, Victoria, Australia; University of Missouri, UNITED STATES

## Abstract

An important feature of a potential vaccine against HIV is the production of broadly neutralising antibodies (BrNAbs) capable of potentially blocking infectivity of a diverse array of HIV strains. BrNAbs naturally arise in some HIV infected individuals after several years of infection and their serum IgG can neutralise various HIV strains across different subtypes. We previously showed that vaccination of cows with HIV gp140 AD8 trimers resulted in a high titre of serum IgG against HIV envelope (Env) that had strong BrNAb activity. These polyclonal BrNAbs concentrated into the colostrum during the late stage of pregnancy and can be harvested in vast quantities immediately after calving. In this study, we investigated the effect of prolonged HIV gp140 vaccination on bovine colostrum IgG HIV Env-binding and BrNAb activity over subsequent pregnancies. Repeated immunisation led to a maintained high titre of HIV Env specific IgG in the colostrum batches, but this did not increase through repeated cycles. Colostrum IgG from all batches also strongly competed with sCD4 binding to gp140 Env trimer and with human-derived monoclonal VRC01 and b12 BrNAbs that bind the CD4 binding site (CD4bs). Furthermore, competition neutralisation assays using RSC3 Env gp120 protein core and a derivative CD4bs mutant, RSC3 Δ371I/P363N, showed that CD4bs neutralising antibodies contribute to the neutralising activity of all batches of purified bovine colostrum IgG. This result indicates that the high IgG titre/avidity of anti-CD4bs antibodies with BrNAb activity was achieved during the first year of vaccination and was sustained throughout the years of repeated vaccinations in the cow tested. Although IgG of subsequent colostrum batches may have a higher avidity towards the CD4bs, the overall breadth in neutralisation was not enhanced. This implies that the boosting vaccinations over 4 years elicited a polyclonal antibody response that maintained the proportion of both neutralising and non-neutralising CD4bs antibodies.

## Introduction

Viral infections continue to pose a great strain on global health, especially persistent viruses such as HIV that can rapidly mutate to escape the immune control and antiviral drugs. Access to anti-retroviral therapy for HIV infected individuals has been effective in reducing the number of new HIV infections, particularly in poor and middle-income countries. In addition, some of these anti-viral chemotherapeutics have demonstrated utility in pre-exposure prophylaxis (PrEP). However, poor drug bioaccumulation and retention in the female genital tract indicates a need for more durable preventative strategies such as a broadly effective HIV vaccine eliciting high levels of mucosal BrNAb and/or a long acting directly installed HIV microbicides that can be used without the consent or knowledge of a sexual partner. The efficacy of systemic delivery of PrEP, such as daily administration of oral tenofovir, has been relatively unsuccessful in women [1] compared to the efficacy in preventing transmission to men due to relatively poor pharmacokinetic and pharmacodynamics challenges that these drugs encounter in vaginal tissues [2].

Microbicides are drug and/or non-drug products that are directly installed onto the genital mucosae that inhibit most sexually transmitted infections. The first products tested utilised the surfactant-containing spermicides in a large Phase III trial [3] but soon after, evidence showed that these compounds also disturb the epithelial barrier and increase the viral infectivity [4]. Though acid-buffering gels were hypothesised to lower vaginal pH and inactivate HIV virus [5], it was shown that their potency is insufficient to avoid infection. Also, long chain polyanionic compounds failed in clinical trials [6]. The current microbicide focus is on more potent antiretroviral (ARV) products such as the nucleotide reverse transcriptase inhibitor, tenofovir gel, which was 39% effective at preventing HIV acquisition in the CAPRISA 004 trial [7]. However, affordability and accessibility for poor countries, poor tissue retention, and the potential selection of viral drug-resistance makes the use of drug-based microbicides difficult. Combination of microbicides that include BrNAbs may be a more effective preventative strategy. Production of high level of BrNAbs in immunised bovine colostrums [8] could be a promising and cheap resource for development of combination microbicides.

Despite three decades of research, the ambitious goal of an effective HIV vaccine has not been fully achieved, even though various Env proteins that bind BrNAbs have been extensively trialed in animal models and to a lesser extent, humans. Although many candidate Env immunogens generate high Env-binding titres, most of these antibodies neutralise only sensitive strains or viruses that match the immunogen. In addition, even after repeated boosting, the desirable breadth of neutralisation has not been achieved [9, 10].

Elicitation of BrNAbs in HIV infected individuals usually occurs late during infection, and typically requires several months to years to appear [11]. Although these antibodies were initially considered rare in patients, recent studies demonstrated that almost 25% of HIV positive sera are capable of neutralising many circulating virus strains. More interestingly, 10% of the mentioned sera have neutralisation activity against most common HIV strains. BrNAbs are capable of neutralising a broad range of recently transmitted HIV variants since they usually target the conserved regions of the virus [12]. BrNAbs are able to bind native viral spikes while most of the antibodies raised against Env immunogens are directed to the gp41 and/or monomeric gp120 regions that are not exposed on the native and mature trimeric virus spikes [13].

To date, vaccination against HIV in human and animal models have yielded no or only narrow neutralising antibodies, respectively. However, recent studies in llamas and cows using HIV uncleaved gp140 as a vaccine antigen have shown more success in eliciting BrNabs. McCoy and colleagues demonstrated that multiple immunisations with recombinant trimeric HIV-1 envelope proteins in a llama can elicit characteristic heavy chain-only antibodies with broad and potent neutralisation activity [14]. We obtained a similar result using HIV gp140 Env to vaccinate cows. Long duration vaccination of cows prior to conception (with additional boosting throughout their pregnancy) elicited high titres of antigen binding antibodies with broad and potent BrNAb activity in both serum and colostrum samples. Colostrum purified IgG from two cows (7004 and 7008) that had the longest duration exposure to the vaccine exhibited anti-CD4 binding site (CD4bs) activity by blocking b12 and VRC01 antibody binding to gp140 [8]. Later, we found that bovine anti-HIV antibodies also have antibody dependent cellular cytotoxicity (ADCC) activity, which may play a critical role in protection against HIV infection [15].

In our study, we aimed to investigate whether a longer vaccination regimen can improve the overall antibody binding titre and neutralisation potency of anti-Env binding antibodies in bovine colostrums samples from cows 7004 and 7008 that had already produced BrNAbs.

## Material and Methods

### Production of AD8 gp140 and vaccination of cows

We previously described the production of experimental HIV vaccines including an oligomeric clade B AD8 gp140 of Env truncated at the membrane proximal external region [16]. In brief, pN1-AD8-140 vector was stably transfected into HeLa cells followed by purification of gp140 trimers from harvested cell culture supernatant using lentil-lectin affinity chromatography and size exclusion chromatography.

**Fig 1:**
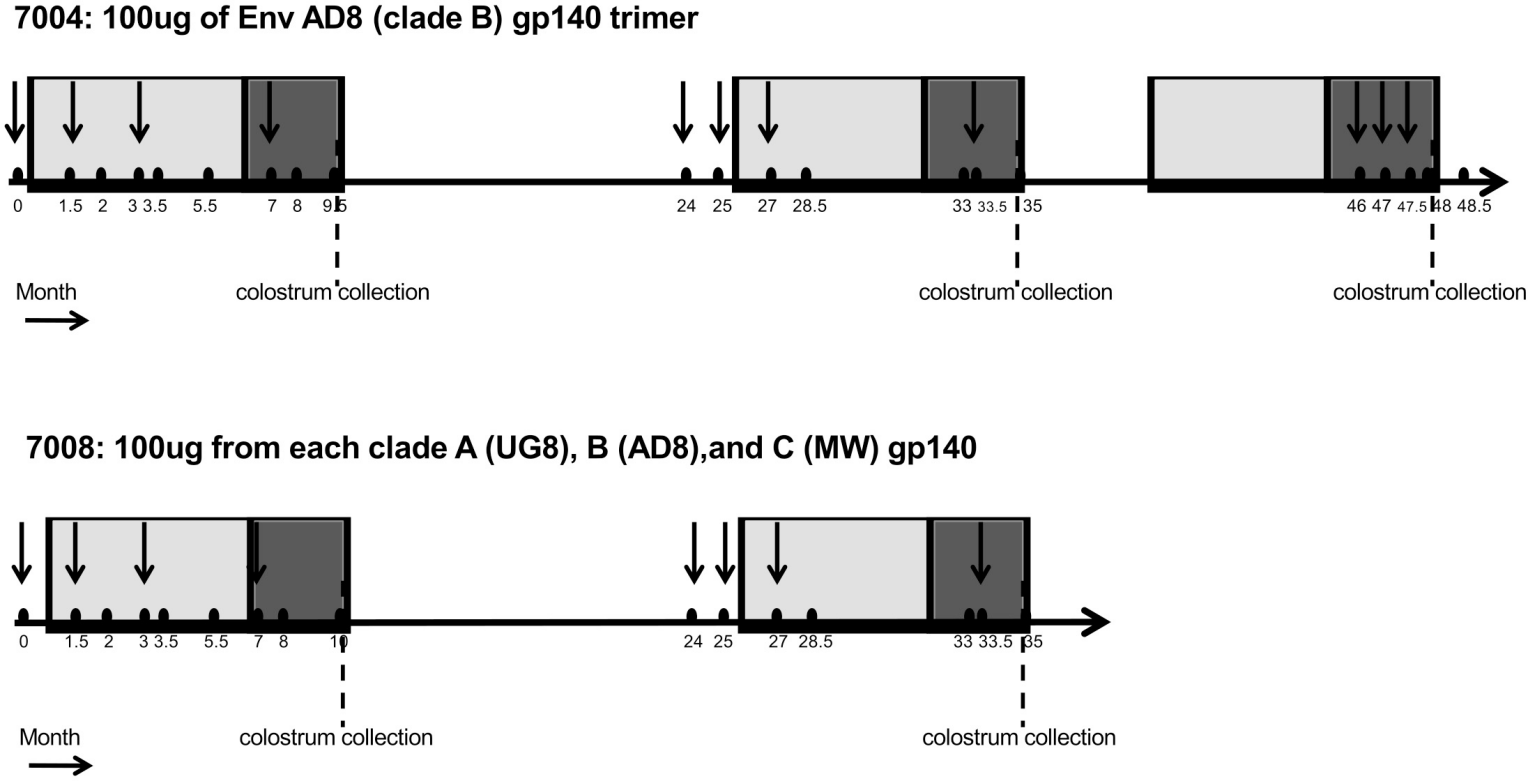
Vaccination regimen. The vaccination was carried out during three pregnancies for 7004 cow and two pregnancies for 7008 cow (shaded in grey). Cow 7004 received 100ug of subtype B uncleaved AD8 gp140 Env protein for all vaccination points. Cow 7008 received a mixture of AD8, MW and UG8 gp140 for the first pregnancy, and AD8 gp140 for the second pregnancy. Two batches of 7008 hyperimmune colostrum and three batches of 7004 hyperimmune colostrum were collected after calving. Arrows indicate boosting vaccinations. Grey box: pregnancy period. Dark box: the third trimester of pregnancy when serum IgG concentrates into the colostrum.

A pair of four-year-old female Holstein Friesian cattle *(Bos Taurus)* who had both successfully delivered a calf were subsequently enrolled into our previously reported year long vaccination and calving study [8]. These animals, called 7004 and 7008, were then enrolled into an ongoing study that was performed on an open-grazing experimental farm for cattle under a Scientific Premises License from the Bureau of Animal Welfare, in the state of Victoria (POCTAA(3)003 A04). Long term repeated vaccinations were conducted with approval from the Immuron Animal Ethics Committee constituted according to the government of Victoria Bureau of Animal Welfare [approval POCTAA (3)003; A04]. Vaccinations contained 100 μg of purified gp140 Env in adjuvant (Montanide ISA 206; Seppic, France) injected intramuscularly into the rear flank of the cows as previously described [8]. While cow 7004 received only AD8 gp140, cow 7008 received a mixture of AD8 (AD8 clone of ADA), MW (93MW965.26 strain) and UG8 (92UG037.8 strain) gp140 (33.33 μg each) (trimix) for the first studied pregnancy [8]. For the ongoing vaccinations described here only AD8 gp140 was administered during the subsequent pregnancies of both 7004 and 7008. In total, cow 7008 had two rounds of vaccination and cow 7004 had 3 rounds of vaccinations over a period of 3–4 years. The vaccination regime is outlined in Fig 1. Each vaccination round consisted of three or four vaccinations. For the primary and secondary round of vaccination gp140 Env was injected before conception in the first, second, and third trimester of pregnancy. For the third round of vaccination of 7004 cow all three injections were only administered during the third trimester. Bovine colostrum samples were collected within six hours postpartum by milking and were stored immediately at −20°C. Cow 7008 was euthanized 40 months into the study after developing a pneumonia unrelated to the vaccination. Cow 7004 was euthanized 6 weeks after her last calving at 50 months into the study after developing mastitis that was unrelated to any vaccination and did not respond to antibiotic therapy. Carcasses were incinerated and no animal products entered the food chain.

### Preparation of colostrum and purified colostrum IgG

The colostrum samples from cows 7004 and 7008 were prepared and purified as described previously [8]. Briefly, bovine colostrum samples were defatted by centrifugation at 10,000 × *g* for 30 min at 4°C, and pasteurised by incubation at 63°C for 30 min, and further centrifugation at 10,000 × *g* for 10 min. The pH of defatted pasteurised colostrum sample was adjusted to 4.6 using 0.2 M sodium acetate solution (pH 4.0) and then mixed at 37°C for 2 h to precipitate the casein and produce colostrum whey. The samples were cooled and centrifuged 10,000 × *g* for 30 min and then the pH of the colostrum whey was adjusted to pH 6.6. The IgG was purified from the colostrum whey using protein G Sepharose chromatography (GE Healthcare) according to the manufacturer’s protocol. Finally, the purified IgG was concentrated and dialysed against phosphate-buffered saline (PBS) using Millipore Amicon Ultracel 30K ultrafiltration membrane units, filter sterilised and concentrations were measured by reading absorbance at 280 nm.

### ELISA and competition ELISAs

Enzyme-linked immunosorbent assay (ELISA) was performed as previously described [8] in order to measure the AD8 gp140-specific IgG titre in serum and colostrum samples. Competition ELISAs against BrNAbs were carried out as previously described using human monoclonal BrNAbs b12 and VRC01 (obtained through the AIDS Research and Reference Reagent Program).

Competition ELISA against the CD4bs epitope on AD8 gp140 Env protein was carried out using sCD4 and polyclonal IgG derived from pre-immune (Batch 0) and all 3 batches of 7004 immune colostrum. In brief, AD8 gp140 (1μg/ml) coated plates were incubated with different concentration of purified IgG from colostrum samples for 2 hours. Then 500ng/ml sCD4 was added to each well and incubated for a further 2 hours. 1/1000 dilution of Anti-CD4 antibody (clone OKT4 Biolegend) was later added and incubated for 2 hours. Finally, after 1 hour incubation with 1/1000 dilution of horse-radish peroxidase (HRP) conjugated Anti-Mouse IgG+A+M (H+L) (Life technologies), colour development was performed using 3,3’-5,5’-tetramethylbenzidine (TMB), and absorbance was measured at 450 nm against a reference of 690 nm.

### Neutralisation assay and competition neutralisation assay

The neutralisation assay using the CF2th/CD4/CCR5/CXCR4 (Cf2th) canine cell line and flow cytometry was performed as previously described [16]. HIV pseudoviruses were produced by co-transfection of full-length Env expression plasmids and an EGFP-expressing proviral reporter plasmid (pNL-4.3ΔenvNef-EGFP). The plasmids used expressed HIV-1 Env from these strains: AD8 (pCMV-AD8; prepared from the pNL.AD8 (AD8) clone of ADA; provided by M. Martin); MN (pSVIII-MN; provided by J. Sodroski); ZM53M.PB12 and ZM135M.PL10a (AIDS Reference Reagent Program (ARRP) contributed by E. Hunter and C. Derdeyn); SC 422661.8 (ARRP contributed by B. H. Hahn and J. F. Salazar-Gonzalez); MuLV (pSVIII-MuLV). Pseudoviruses were collected after 48–72 hours and stored at -80°C. 10μl of virus at an infectivity of 10% was mixed with 20μl of 7004 purified polyclonal colostrum IgG at different dilutions and incubated for 1 hour at 37°C. After incubation time, 70μl of CF2th cells (2 × 10^4^ per well) was added to each well followed by spinoculation at 1200*xg* for 2 hours at room temperature followed by a fresh media change of 200μl. The Infectivity of the pseudoviruses in the presence of antibodies was analysed by flow cytometry using the LSRII (BD Biosciences) and analysed using Flowjo version 9.2. The 50% inhibitory concentration (IC_50_) was reported as the reciprocal IgG concentration required to inhibit the virus infection by 50%.

Competition neutralisation assay using wild-type resurfaced stabilised core (RSC3) HIV-1 envelope core recombinant protein, or the variant containing CD4 binding site knockout mutations (RSC3 Δ371I/P363N), were performed as described previously [17, 18]. Briefly, a final concentration of 25μg/ml of RSC3 Env gp120 protein core or RSC3 Δ371I/P363N mutant or PBS was incubated with 7004 purified polyclonal colostrum IgG serially diluted 2-fold from 1000μg/ml for 30 min at 37°C before the addition of MN pseudovirsus. Competition between colostum IgGs and the core proteins was determined by calculating the area under the curve (AUC) in the presence or absence of competing protein using Prism 7.0 (GraphPad, San Francisco, CA). The percent contribution of CD4bs antibodies to overall colostrum IgG neutralising activity was calculated as 100% × (AUC without protein − AUC with protein)/(AUC without protein) [19]. In addition, as a complementary analysis we calculated the neutralisation IC_50_ in the presence of RSC3 Env gp120 protein core and RSC3 Δ371I/P363N mutant to determine the percentage of RSC3 IC_50_ increase relative to the IC_50_ of RSC3 Δ371I/P363N.

## Results

### Level of HIV- specific IgG in purified colostrum IgG

**Fig 2:**
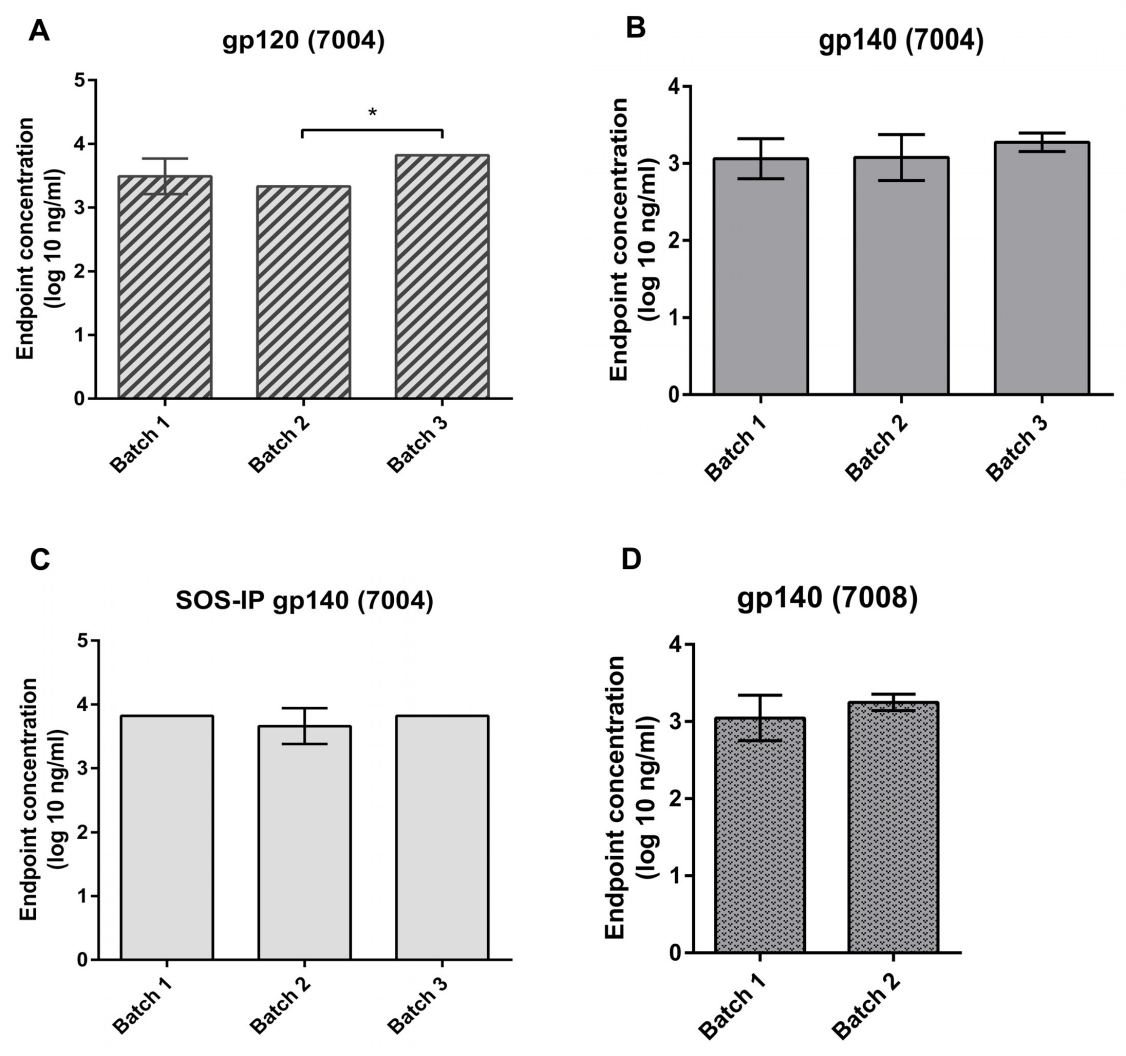
AD8 Env-specific titre of purified IgG from colostrum samples of 7004 and 7008 cows. The cows received a prolonged vaccination. Antigen binding activity and endpoint concentrations of anti-Env IgG from the three batches of 7004 colostrum was tested against A) cleaved gp120 monomer B) uncleaved gp140 oligomer and C) cleaved SOS-IP gp140 trimer. D) The two batches of 7008 purified IgG from colostrum samples were tested only against uncleaved gp140 oligomer. Reciprocal IgG endpoint concentration was measured by ELISA. A positive signal was determined as an optical density (OD) of >2-fold of pre-immune (Batch 0) colostrum purified IgG. The results represent the mean of 3 replicates (7004-SOS-IP gp140), 4 replicates (7004-gp140), 6 replicates (7004-gp120), 4 replicates (7008-gp140) and error bars represent standard deviation (SD).

In the current study, we looked whether prolonged vaccination with oligomeric gp140 Env resulted in a sustained high titre of HIV-specific IgG in colostrum samples. ELISA assay results showed that the endpoint concentration of anti-AD8 gp120 Env antibodies in 7004 cow was 3.49 ± 0.28 and 3.33 log_10_ ng/ml for the first and second batch, respectively while it increased to 3.82 log_10_ ng/ml for the third batch (Fig 2A). The endpoint concentration of anti-AD8 gp140 Env in 7004 cow was consistent in different batches of colostrum: 3.06 ± 0.26, 3.07 ± 0.29 and 3.27± 0.11 log_10_ ng/ml for the first, second and third batch, respectively (Fig 2B).

We also determined the anti-SOS-IP AD8 gp140 Env- IgG titres of 7004 colostrum batches. Endpoint concentrations were very similar to those against AD8 gp140 Env: 3.82 log_10_ ng/ml, 3.66 ± 0.28 log_10_ ng/ml, 3.82 log_10_ ng/ml for first, second and third batch, respectively (Fig 2C). In 7008 cow, the endpoint concentration of anti-AD8 gp140 Env was consistent in two batches of colostrum: 3.04 ± 0.29 and 3.24 ± 0.10 log_10_ ng/ml for the first and second, respectively (Fig 2D). In summary, we observed a high antigen binding activity towards AD8 gp140 < gp120 < SOS-IP gp140 Env protein. However, no significant increase or boosting effect was observed after any additional vaccination.

### Competition of BrNabs and colostrum IgG against the CD4 binding site (CD4bs)

**Fig 3:**
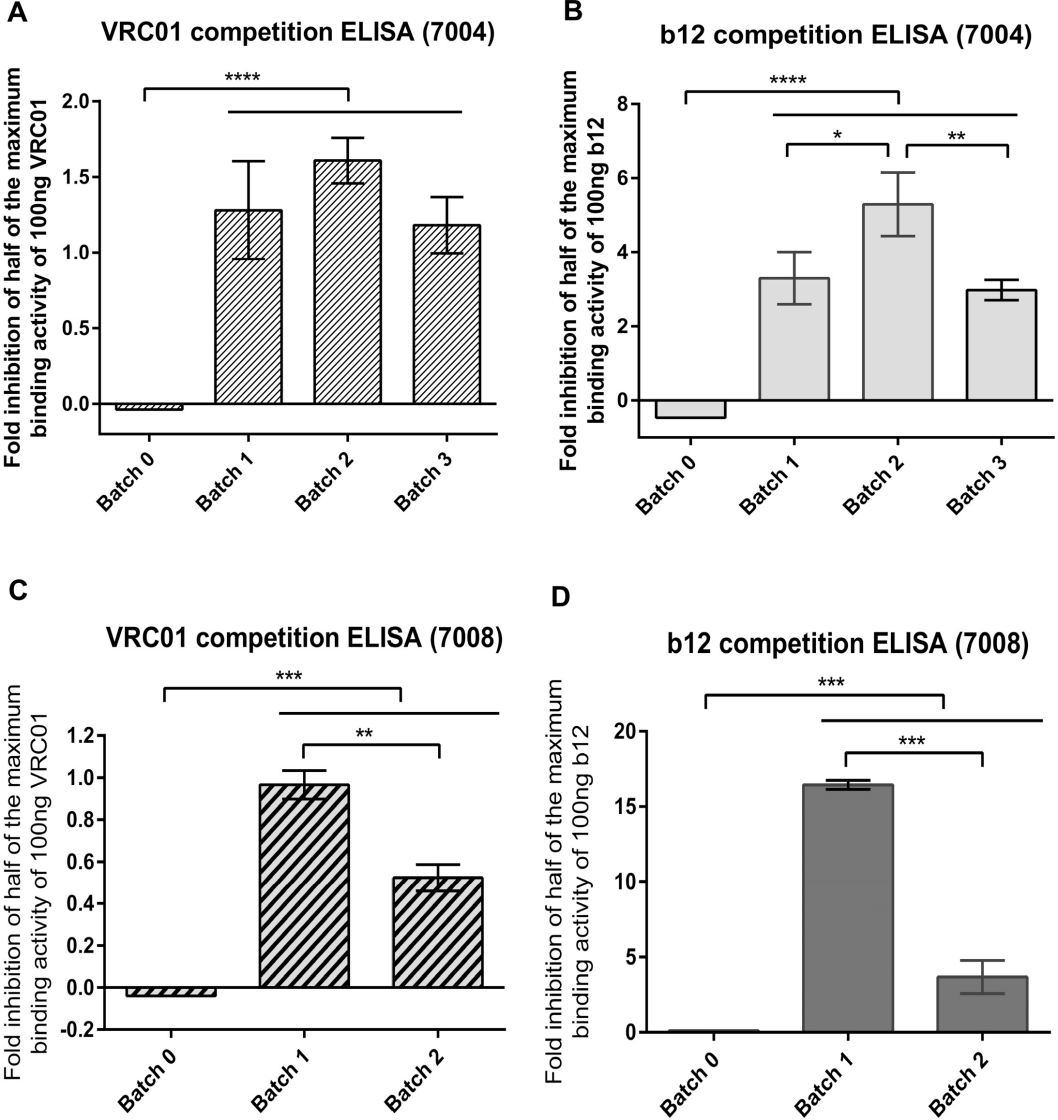
Inhibition of BrNAbs VRC01 and b12 binding to CD4bs epitopes by colostrum IgG from cow 7004 and 7008. A) 7004 competition ELISA against VRC01, B) 7004 competition ELISA against b12, C) 7008 competition ELISA against VRC01, D) 7008 competition ELISA against b12. Competition ELISAs were performed by titrating the human neutralising mAb VRC01 and b12 against a constant background of 100 μg 7004 or 7008 purified colostrum IgG. The binding of VRC01 and b12 mAbs was detected using anti-human HRP conjugated antibody. The inhibition activity was determined by comparison of the amount of mAb VRC01 or b12 that showed the identical optical density to that of half of the maximum binding of 100 ng VRC01 or b12. Batch 0 is the pre-immune collected colostrum sample. Results represent the mean of 3 repeats and error bars show SD values. *P* values were calculated using one-way Anova followed by a Tukey HSD test post-test (****P < 0.0001, ***P < 0.001; **P ≤ 0.01; *P ≤ 0.05).

We have previously shown that cows 7004 and 7008 vaccinated with uncleaved gp140 produce antibodies targeting some of the same Env epitopes than human BrNAbs [8]. Bovine colostrum antibodies inhibited the binding to gp140 Env of BrNAbs VRC01 and b12. The current study aimed to determine whether these desirable antibodies were continuously produced over a long vaccination period (3–4 years) and whether antibodies targeting other neutralising epitopes emerged in the later colostrum batches. A competition ELISA was performed with a single concentration of purified colostrum IgG of 1mg/ml. Consistent with our previous results, 7004 purified colostrum IgG from batch 2 and 3 inhibited the binding of CD4bs targeting antibodies VRC01 and b12 (Fig 3). The fold inhibition of half of the maximum binding activity of 100ng VRC01 for first, second and third batch of 7004 purified IgG was 1.28 ± 0.32, 1.61 ± 0.15 and 1.18 ± 0.18, respectively. As a negative control, VRC01 binding was assessed in the presence of pre-immune colostrum IgG (batch 0) and resulted in -0.03 demonstrating no effect on the binding of VRC01. A significant increase in the inhibition activities of purified IgG samples was observed in 7004 second batch (*P* < 0.0001, One-way Anova test, Fig 3A). In comparison to batch 0 (-0.46 fold inhibition activity), b12 binding was also blocked with significantly greater potency with the different immune colostrum batches (*P* < 0.0001 One-way Anova test, Fig 3B). Similar to VRC01, the first and third batch of purified polyclonal IgG from 7004 colostrum inhibited half of the maximum binding activity of 100ng b12 with 3.30 ± 0.7 and 2.98 ± 0.27, respectively. The highest inhibition binding was achieved from the second batch (5.30 ± 0.86, Fig 3B). Interestingly, the blockage of b12 binding activity to AD8 gp140 with 7004 purified colostrum IgG samples was higher than against VRC01, corresponding to the affinity of these monoclonal BrNAbs. In addition, the purified polyclonal IgG from colostrum samples did not inhibit 2F5 and 2G12 monoclonal BrNAbs that bind the MPER and glycan epitopes on the gp140 trimers (data not shown).

Inhibition of half of the maximum binding activity of 100ng VRC01 in 7008 purified IgG from colostrum samples was 0.96 ± 0.06 and 0.52 ± 0.06 fold for the first and second batch, respectively. The pre-immune batch (Batch 0) inhibition against VRC01 binding was -0.04. Also, 7008 purified IgG of first batch inhibition of half of the maximum binding activity of 100ng b12 binding as 16.43 ± 0.29 fold whereas this value decreased to 3.67 ± 1.09 fold for the second batch. The pre-immune batch (Batch 0) blockade against b12 binding was 0.11 fold. VRC01 and b12 bindings were also blocked with greater potency with 7008 immune colostrum batches compared to the 7008 pre-immune batch (Batch 0), and the profile of 7008 polyclonal antibodies against VRC01 and b12 epitopes is decreased over the time (Fig 3C and 3D).

In conclusion, prolonged vaccination with gp140 Env resulted in the production of bovine colostrum IgG with high-affinity to the CD4bs that is also targeted by BrNAbs VRC01 and b12. In addition, the titre or avidity of bovine polyclonal antibodies against CD4bs epitopes of the mentioned BrNAbs was achieved during the first year of vaccination and sustained throughout the years of repeated vaccinations in the tested cows.

### Purified IgG from colostrum samples inhibit binding of soluble CD4 (sCD4) to CD4bs on gp140 Env protein

**Fig 4:**
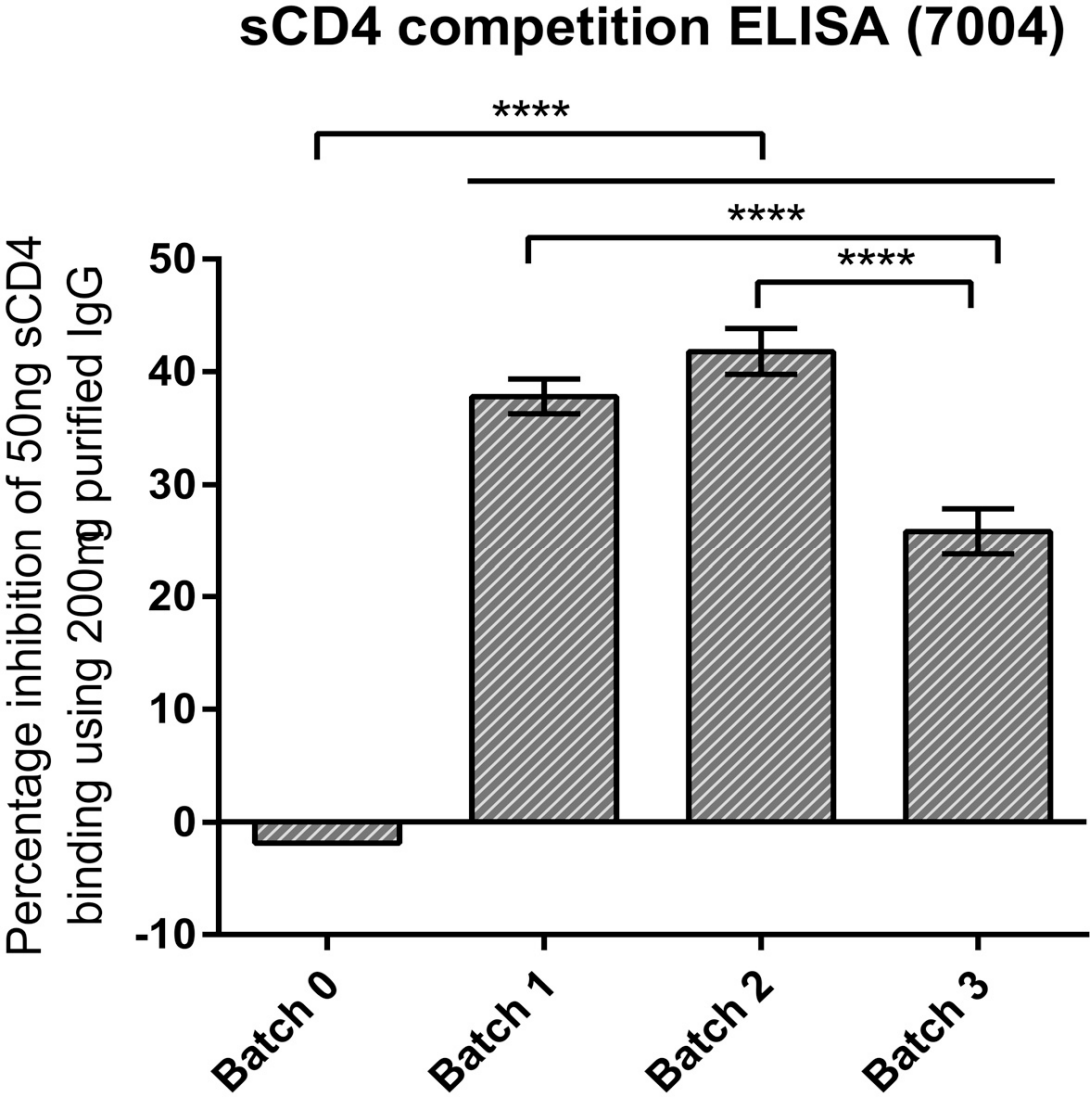
Inhibition of sCD4 binding activity to CD4bs epitope on AD8 gp140 Env by colostrum IgG from cow 7004. The competition ELISA was done using serial concentrations of colostrum purified IgG with the constant amount of 500ng/ml sCD4. Binding of sCD4 was detected using anti-CD4 antibody. Batch 0 is the pre-immune collected colostrum samples. Results represent the mean of 3 repeats and error bars show SD values *P* values were calculated using one-way Anova followed by a Tukey HSD test post-test (****P < 0.0001).

In order to investigate whether the inhibition of BrNAbs VRC01 and b12 binding activity with purified polyclonal IgG from colostrum samples is due to the direct binding of polyclonal antibodies to the CD4bs on gp140 Env, sCD4 competition ELISA was undertaken. The result revealed that the first batch of bovine colostrum antibodies inhibited binding of 50ng sCD4 to AD8 gp140 Env by 37.8% ± 1.53% (Fig 4). This increased to 41.8% ± 2.03 for the second batch demonstrating the highest inhibition activity against sCD4 binding compared to the other two batches. The third batch inhibited the binding of sCD4 by 25.81% ± 2.03%. The pre-immune batch (Batch 0) of colostrum was used as a negative control showing-1.85% inhibition of 50ng sCD4 binding.

### Neutralisation activity of bovine polyclonal IgG over extended vaccination duration

In this study, we assessed the neutralisation activity and potency of purified IgG from different colostrum batches of the 7004 IgG formed in response to the same AD8 gp140 vaccine using a panel of pseudoviruses: MN (Clade B, tier 1), SC422661, clone B (SVPB8) (Clade B, tier 2), ZM53M.PB12, SVPC11 (Clade C, tier 2), ZM135M.PL10a, SVPC15 (Clade C, tier 2) and AD8 (Clade B, immunogen). These data tested for any broadening of the neutralisation activity in response to a single vaccine Env gp140. The neutralisation activity of the purified immunised colostrum IgG against each pseudovirus was calculated by subtracting the neutralisation background of pre-immune colostrum IgG batch (Batch 0). Finally, IC_50_ of each batch was calculated and a positive neutralisation was defined as IC_50_ of ≤2 fold of IC_50_ against MuLV (data not shown). One-way Anova test on log_10_ IC_50_ values was performed followed by Tukey HSD post-test to compare the neutralisation activity of colostrum batches against different pseudoviruses. As shown in Table 1, the IC_50_ and IC_80_ of purified colostrum IgGs were below 0.5μg/μl and 1 μg/μl, respectively, against MN pseudoviruses. Neutralisation against MN did not change significantly through the long vaccination regimen. The IC_50_ values of purified bovine colostrum IgGs against all tested pseudoviruses were below 1ug/μl whereas the IC_80_ values were between 1–4 ug/μl. The IC_80_ of second batch of bovine purified IgG against ZM53M.PB12.SVPC11 pseudovirus (2.29 ± 0.06) was indicated slightly greater IgG levels than those in the first (1.32 ± 0.23) and third (1.62 ± 0.03) batches were required, but these differences were not significant. Although we detected some fluctuations in the neutralisation activity, the three batches were able to demonstrate enduring cross-clade neutralisation activity against selected tier 1, 2 and 3 pseudoviruses: AD8, MN, SC422661, ZM53M.PB12 and ZM135M.PL10a for all batches of colostrum IgG.

**Table 1:** HIV neutralisation profile of 7004 purified bovine colostrum IgG from three different batches.

	Clade	IC_50_ (μg/μl)			IC_80_ (μg/μl)			p value for IC_50_			p value for IC_80_		
		Batch 1	Batch 2	Batch 3	Batch 1	Batch 2	Batch 3	#1 vs #2	#1 vs #3	#2 vs #3	#1 vs #2	#1 vs #3	#2 vs #3
**Tier 1**													
**MN**	B	0.21 ± 0	0.2± 0.01	0.31 ± 0.02	0.57 ± 0.01	0.57 ± 0.04	0.79 ± 0.01	NS	<0.05	<0.05	NS	<0.05	<0.05
**Tier 2**													
**SC422661, clone B (SVPB8)**	B	0.42 ± 0	0.70 ± 0.16	0.39 ± 0.04	1.92 ± 0.29	4.96 ± 0.56	3.18 ± 1.22	NS	NS	NS	NS	NS	NS
**ZM53M.PB12, SVPC11**	C	0.53 ± 0.04	0.57 ± 0.12	0.45 ± 0	1.32 ± 0.23	2.29 ± 0.06	1.62 ± 0.03	NS	NS	NS	<0.5	NS	<0.5
**ZM135M.PL10a, SVPC15**	C	0.74 ± 0.02	0.71 ± 0.16	0.78 ± 0.12	2.17 ± 0.13	1.84 ± 0.9	2.07 ± 0.3	NS	NS	NS	NS	NS	NS
**Immunogen**													
**AD8**	B	0.87 ± 0.02	0.98 ± 0.14	0.81 ± 0.08	2.91 ± 0.5	4.55 ± 0.22	1.99 ± 0.35	NS	NS	NS	NS	NS	<0.5

A positive neutralisation was defined as IC_50_ of ≤2 fold of IC_50_ against MULV

### Evolution of CD4bs antibodies response

**Fig 5:**
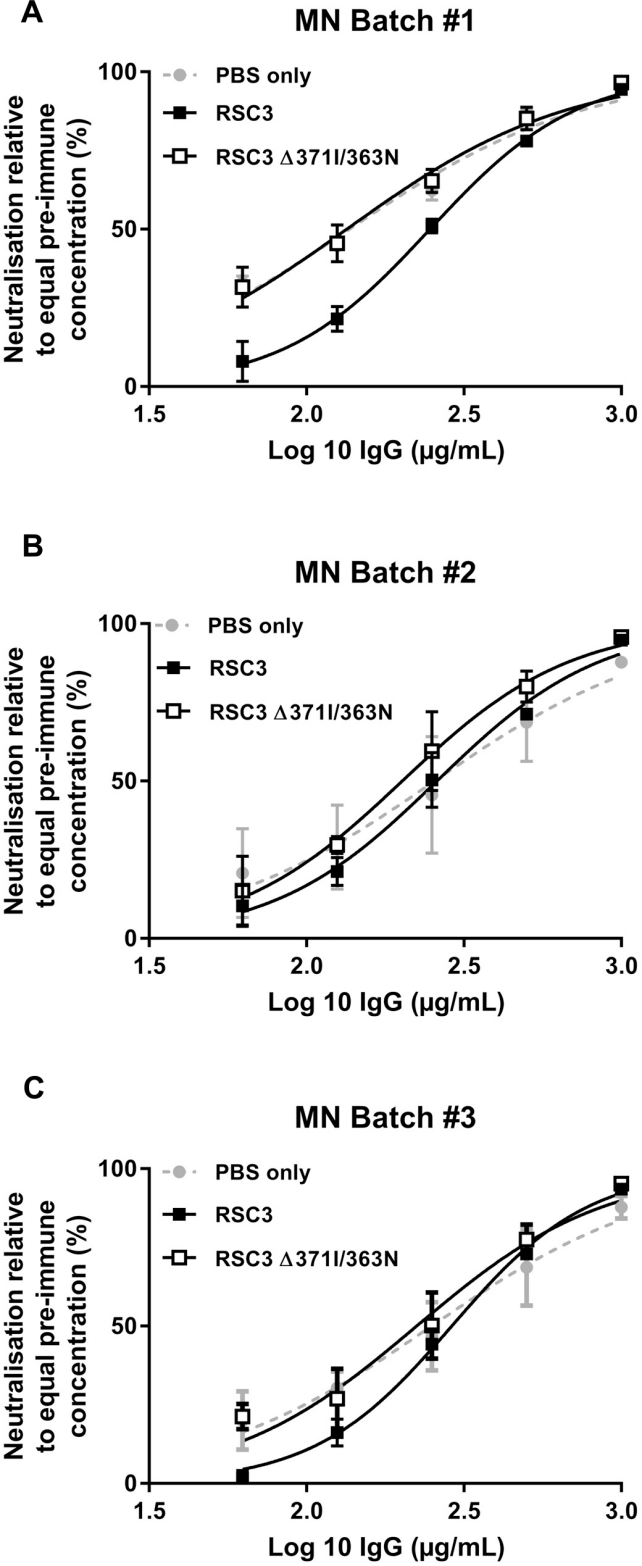
Analysis of 7004 purified polyclonal colostrum IgG CD4bs-directed neutralisation. The neutralisation of purified IgGs against MN pseudovirus was tested in the presence of RSC3, RSC3 Δ371I/P363N or PBS. A) Neutralisation of batch 1, B) Neutralisation of batch 2 and C) Neutralisation of batch 3. Two independent experiments were performed and an average neutralisation was taken.

As summarised in Table 2, the different batches of bovine colostrum inhibited sCD4 or CD4bs mAbs binding to CD4bs epitopes in competition ELISA assays. However, to determine whether the neutralisation of 7004 purified IgGs was mediated by CD4bs neutralising antibodies and maintained over the long vaccination regimen, RSC3 Env gp120 protein core was used in a competition neutralisation assay. RSC3 is engineered to prominently display the CD4-binding site. RSC3 Δ371I/P363N has two mutations that dramatically diminish the binding of CD4bs mAbs to this isogenic gp120 [17, 18]. As shown in Fig 5, RSC3 Env gp120 protein core decreased the neutralisation activity of all 7004 purified bovine colostrum IgG batches against MN pseudovirus (Fig 5A–5C). We examined the differences in the RSC3% AUC reduction compared to RSC3 Δ371I/P363N %AUC. This showed that the competitive inhibition of RSC3 Env gp120 protein core on the neutralisation of 7004 purified bovine colostrum IgG for reporter virus with HIV-1 MN strain Env was greatest for batch 1 (12.5%) while further vaccination led to slightly decreasing values of 9.10% and 7.49% for batch 2 and 3, respectively (Table 2). Furthermore, analysis of neutralisation showed greater IC_50_ in the presence of RSC3 Env gp120 for all batches compared with neutralisation IC_50_s in the presence of RSC3 Δ371I/P363N. The highest increase of RSC3 IC_50_ relative to RSC3 Δ371I/P363N IC_50_ (84.10%) was obtained with the first batch of colostrum whereas this figure was decreased to 28.80% and 29.40% for the second and third batches, respectively. The AUC% and IC_50_ analyses both show that all batches contain CD4bs neutralising bNAbs, but these become a smaller component of the neutralising activity in later batches (Table 2).

**Table 2:** A summary of anti-HIV antibody profile of bovine colostrum batches in competition ELISA and neutralisation assays.

	Fold inhibition of VRC01 binding to AD8 gp140 Env	Fold inhibition of b12 binding to AD8 gp140 Env	% Inhibition of sCD4 binding to AD8 gp140 Env	NeutralisationIC_50_	RSC3%AUC reduction compared to RSC3 Δ371I/P363N	Neutralisation IC_50_ in the presence of RSC3	Neutralisation IC_50_ in the presence of RSC3 Δ371I/P363N	RSC3% IC_50_increase relative to RSC3 Δ371I/P363N
**Batch 1**	1.28 ± 0.32	3.30 ± 0.70	37.8 ± 1.53	0.21 ± 0	12.50	0.24	0.13	84.10
**Batch 2**	1.61 ± 0.15	5.30 ± 0.86	41.8 ± 2.03	0.20 ± 0.01	9.10	0.25	0.20	28.80
**Batch 3**	1.18 ± 0.18	2.98 ± 0.27	25.81± 2.03	0.31 ± 0.02	7.49	0.28	0.22	29.40

IC_50_ values are presented in μg/μl.

## Discussion

A major goal is to control the AIDS epidemic through the implementation of a vaccine which can protect the individuals prior to the infection by generating BrNAbs. Induction of neutralising antibodies via immunisation has been an insurmountable challenge in the initial vaccine field. Though high level of anti-Env binding has been achieved, neutralisation has been limited [20, 21]. Naturally, elicitation of cross-reactive BrNAbs takes years and is restricted to 10–30% of HIV infected human individuals [11, 22–25] which also depends on the strain of Env (one of the major factors which impacts on the induction of BrNAbs) [26, 27]. Kramski and colleague’s results from bovine vaccination showed that the AD8 gp140 could elicit BrNAbs after immunisation [8], so we aimed to investigate the effect of a prolonged vaccination on the development of BrNAbs.

According to the previous studies, in addition to the strain of Env protein, the form of immunogen plays a critical role in the success of a vaccine regimen. During natural HIV-1 infection, Env is presented to the human humoral immune system in various forms, but importantly as functional Env trimers on the surface of virions and infected cells, together with the disassembled or conformationally open Env trimers, shed monomeric gp120 and residual gp41 stumps and aggregated gp160 from the infected cells [28, 29]. The relative quantity of functional Env trimers may be limited than non-functional gp120 molecules that are more frequent on the surface of infectious HIV-1 virions, as well as on any shed Env. Also, the antigenic epitopes are less accessible on native form of Env compared with gp120 monomers. This can lead to the production of antibodies that can capture Env protein that is unable to neutralise the virion [29]. An efficient vaccination needs to elicit antibodies that bind to functional trimer Env protein [30–32]. Immunisation with trimeric Env has resulted in effective neutralising antibodies in sera compared with the vaccination with monomeric counterparts demonstrating the importance of well-designed Env for vaccination [33–37]. In this study we aimed to investigate the effect of further immunisations with AD8 trimeric uncleaved gp140 on the production of Env specific antibodies and BrNAbs in two cows that previously responded with BrNAb production after one vaccination regimen. While both cows responded with sustained titre of antibodies against uncleaved gp140 immunogen, we pursued the question of whether ongoing vaccination of cow 7004 with the same immunogen would therefore increase the breadth of reporter pseudovirus neutralisation.

Measurement of anti-Env activity, which is a combination of neutralising and non-neutralising antibodies is the first step to evaluate a vaccine success. Though most HIV research focus is on BrNAbs, the result of RV144 vaccine study showed a 31% reduction in infection rate despite the lack of detectable HIV neutralising antibodies [38]. Recent analysis demonstrated that low risk of infection in the presence of non-neutralising antibodies correlates with V1 V2 variable loop binding [39] and those antibodies can mediates ADCC activity [40]. In the current study, the results showed that repeated vaccination with trimeric Env AD8 gp140 induced a sustained high level of anti-Env antibodies against AD8 trimeric gp140 and monomeric gp120 but did not enhance this response.

In addition, we showed that immunisation with oligomeric AD8 gp140 induced IgG against the SOS-IP gp140 Env form. To date, the closest natural structure to the functional Env protein is SOS-IP Env which is a recombinant stabilised form on which most non-neutralising epitopes are obstructed or hidden [41–44]. Therefore, the binding of colostrum IgG to SOS-IP Env demonstrates that vaccination with oligomeric AD8 gp140 Env was efficient at eliciting antibodies against the epitopes exposed on SOS-IP Env. Moreover, the IgG titre against HIV AD8 SOS-IP Env was constant over the whole vaccination regimen, which was in agreement with binding antibodies to other Env forms and suggests that one vaccination cycle is sufficient to induce high level of anti-Env binding antibodies.

We previously showed that the cow vaccinated with AD8 gp140 produced colostrum antibodies against the CD4bs epitope [8]. Lynch and colleagues showed that the development of CD4bs antibodies such as VRC01-like antibodies require a few years of maturation to mediate the detected neutralisation [18]. We therefore attempted to enhance the level of CD4bs binding by a prolonged vaccination in cow 7004 and 7008. VRC01/b12 competition ELISAs showed that inhibition activity of 7004 and 7008 polyclonal IgG from colostrum samples against CD4bs epitopes of the mentioned BrNAbs was achieved during the first year of vaccination and sustained throughout the years of repeated vaccinations in the tested cows.

In our study, the neutralisation profile was in agreement with the binding activity of colostrum IgG demonstrating that the first vaccination cycle was efficient enough to induce BrNAbs. However, a prolonged vaccination regimen demonstrated enduring cross-clade neutralisation activity against selected tier 1, 2 and 3 pseudoviruses (AD8, SC422661, ZM53M.PB12 and ZM135M.PL10a) with no significant increase in neutralisation potency and breadth. While neutralisation activity was maintained over the second vaccination regimen, it decreased during the third. Unfortunately, the increased titre and binding activity of CD4bs antibodies in the second batch did not enhance cross-clade neutralisation potency. The competition neutralisation assay with RSC3 Env gp120 protein core and RSC3 Δ371I/P363N mutant showed that CD4bs neutralising antibodies contribute to the neutralising activity of purified bovine colostrum IgGs. RSC3 competition neutralisation assay showed that the first batch had the highest bovine CD4bs neutralising antibody titre. The enduring titre of neutralising antibodies without any significant changes among the batches in neutralisation assay, suggests that the bovine immune system had undergone affinity maturation toward other neutralising epitopes on HIV Env rather than CD4bs. In addition, the increase of VRC01, b12 and sCD4 binding inhibition activity of the second batch may be due to the stimulation of antibodies that bind to the epitopes adjacent to the CD4bs that sterically occluded access to the mentioned region.

In conclusion, prolonged vaccination sustained AD8 soluble gp140, gp120 and SOS-IP gp140 antibody titre as well as CD4bs site antibodies. We were also able to demonstrate durable cross-clade neutralisation activity against selected tier 1, 2 and 3 pseudoviruses. However, it is curious why bovine immunisation yields such high titers of BrNAbs after the first course of vaccination that does not occur following vaccination of other species. Vaccinations with HIV Env that has evolved to evade human antibody immunity may present epitopes in bovine that are anergic in primates [16]. Also, it is shown that vaccination of individuals with gp41 subunit vaccine resulted in mostly non-neutralising antibodies which reacted with intestinal microbiota. This demonstrates that preexisting immunity to microbial community may mislead the immune system to an unproductive target [45]. So, it is also possible that the microbiota that shape the polyfunctional B-cell precursors that spawn BrNAbs in humans are different in a ruminant, like the bovine, leading to a more rapid and reproducible production of neutralising antibodies. Finally, the unprecedented long length of the CDRH3 regions of the bovine IgG [46, 47] lead to V-regions that make long CDRH3 with an average CDRH3 length that is equal to the maximum size for human CDRH3 frequently observed in BrNAbs from human [48, 49]. Also, high rate of somatic mutation in bovine antibodies is a common feature [50] whereas the human immune system requires a prolonged continuous antigen exposure to elicit BrNAbs with characteristic highly mutated and long CDRH3. At the molecular level, sequence analysis and 3D structural modeling of the bovine anti-HIV antibodies in future studies might give us an insight into better understanding of the critical features for HIV Env binding and/or neutralisation activity.
